# CARDIOVASCULAR DISEASE RISK SCORES AND INCIDENT DEMENTIA AND COGNITIVE DECLINE IN OLDER MEN AND WOMEN

**DOI:** 10.1093/geroni/igad104.2400

**Published:** 2023-12-21

**Authors:** Swarna Vishwanath, Ingrid Hopper, Andrew Tonkin, Anne Murray, Raj Shah, Robyn Woods, John McNeil, Joanne Ryan

**Affiliations:** Monash University, Melbourne, Victoria, Australia; Monash University, Melbourne, Victoria, Australia; Monash University, Melbourne, Victoria, Australia; Hennepin HealthCare and University of Minnesota, Minneapolis, Minnesota, United States; Rush University Medical Center, Chicago, Illinois, United States; Monash University, Melbourne, Victoria, Australia; Monash University, Melbourne, Victoria, Australia; Monash University, Melbourne, Victoria, Australia

## Abstract

**Background:**

Risk factors for cardiovascular disease (CVD) also increase the risk of dementia. However, whether CVD risk scores are associated with dementia risk in older adults remains unclear. The aim of this study was to determine whether CVD risk scores are associated with dementia and cognitive decline in older men and women.

**Methods:**

19,114 participants from a prospective cohort of individuals aged 65+ years. The Atherosclerotic Cardiovascular Disease risk score (ASCVDRS), Systematic Coronary Risk Evaluation 2 – Older Persons (SCORE2-OP) and the Framingham risk score (FRS) were calculated at baseline. Risk of dementia (DSM-IV criteria) and cognitive decline (>1.5 standard deviation (SD) decline from previous assessment in cognitive function) were assessed using hazard ratio.

**Results:**

Over a median follow-up of 6.4 years, 850 individuals developed dementia and 4352 cognitive decline. Men and women in the highest ASCVDRS tertile had a 41% (95%CI 1.08,1.85) and 45% (1.11,1.89) increased risk of dementia compared to the lowest tertile respectively. Likewise, men and women in the highest SCORE2-OP tertile had a 64% (1.24,2.16) and 60% (1.22,2.11) increased risk of dementia. Findings were similar but the risk was slightly lesser when examining risk of cognitive decline for both ASCVDRS and SCORE2-OP. FRS was only associated with the risk of cognitive decline among women (highest vs. lowest tertiles: 1.13 [1.01-1.26]).

**Conclusion:**

These findings suggest the utility of CVD risk scores in clinical practice, to not only assess future risk of CVD, but also as potential early indicators of cognitive impairment in older men and women.

